# Differences in spinal posture and mobility between children/adolescents with obesity and age-matched normal-weight individuals

**DOI:** 10.1038/s41598-022-19823-z

**Published:** 2022-09-16

**Authors:** M. E. Bayartai, C. E. Schaer, Hannu Luomajoki, G. Tringali, R. De Micheli, A. Sartorio

**Affiliations:** 1 Institute of Physiotherapy, Health Sciences, Zurich University of Applied Sciences ZHAW, Katharina-Sulzer-Platz 9–P.O. Box, 8401 Winterthur, Switzerland; 2 Department of Physical Therapy, School of Nursing, Mongolian National University of Medical Sciences, Ulaanbaatar, Mongolia; 3 Idiag AG, Fehraltorf, Switzerland; 4 Experimental Laboratory for Auxo-Endocrinological Research, Istituto Auxologico Italiano, IRCCS, Piancavallo-Verbania, Italy; 5 Division of Auxology and Metabolic Diseases, Istituto Auxologico Italiano, IRCCS, Piancavallo-Verbania, Italy

**Keywords:** Endocrinology, Rheumatology, Risk factors

## Abstract

The aim of this study was to cross-sectionally explore the association of obesity with spinal posture and mobility, commonly associated with musculoskeletal problems, by comparing the spinal parameters between 90 obese and 109 normal-weight children and adolescents. A non-invasive electromechanical device, the Idiag M360 (Idiag, Fehraltorf, Switzerland), was used to measure the spinal parameters. An age-and-sex-adjusted two-way analysis of variance (ANOVA) was used to determine postural and mobility differences between the two groups. Children and adolescents with obesity had significantly greater thoracic kyphosis [difference between groups (Δ) = 13.0^0^, 95% CI 10.1^0^–15.8^0^, p < 0.0001] and thoracic extension (Δ = 6.5^0^, 95% CI 2.9^0^–11.6^0^, p = 0.005), as well as smaller mobility in thoracic flexion (Δ = 5.0^0^, 95% CI 1.2^0^–8.8^0^, p = 0.01), thoracic lateral flexion (Δ = 17.7^0^, 95% CI 11.6^0^–23.8^0^, p < 0.0001), lumbar flexion (Δ = 12.1^0^, 95% CI 8.7^0^–15.5^0^, p < 0.0001), lumbar extension (Δ = 7.1^0^, 95% CI 3.1^0^–12.2^0^, p = 0.003) and lumbar lateral flexion (Δ = 9.1^0^, 95% CI 5.5^0^–12.8^0^, p < 0.0001) compared to the normal-weight children and adolescents. These findings provide important information about the characteristics of the spine in children and adolescents with obesity and unique insights into obesity-related mechanical challenges that the spine has to withstand and strategies designed to improve spinal mobility in this young population.

## Introduction

The prevalence of obesity among children and adolescents has been increasing across worldwide populations, causing both psychological and physical consequences^1,2^. Between 1975 and 2016, the global prevalence of obesity increased eight-fold in children and adolescents^3^. Obesity can negatively affect various body systems and lead to an increased risk of numerous health conditions, including musculoskeletal conditions, cardiovascular diseases, metabolic syndrome and gastrointestinal and pulmonary conditions^2^. Exercise, motion, diet and behavioural changes still play a crucial role in the management of obesity and obesity-related conditions in paediatric practice compared to medications^2,4,5^. Physical inactivity, reduced body motion and sedentary lifestyle contributing to obesity^2^ are also considered to be modifiable risk factors for common musculoskeletal conditions, such as knee osteoarthritis and low back pain^6–8^.

Obesity appears to alter spinal postures and movement in adult populations^9–11^. Spinal posture and flexibility commonly examined in clinical practice as part of a musculoskeletal assessment, are reported to play an important role in the performance of daily activities, as well as many occupational and recreational tasks. Additionally, alterations in spinal movement are often associated with low back pain^12,13^. Obesity is not only associated with low back pain, which is one of the most common musculoskeletal conditions but it also increases the risk of low back pain^14^. Low back pain is a common problem in school-aged children as well. A subsequent increase in the prevalence of low back pain in children becomes similar to the rates of low back pain in adult populations when they reach 18 years of age^1^. Low back pain is multifactorial and the biophysical aspect is considered one of the factors that substantially contribute to it^15^. For example, alterations in spine mobility may increase abnormal loading that leads to tissue degeneration, potentially resulting in pain and other clinical symptoms^16^. Importantly, prospective studies have shown that restriction in the lumbar range of motion in the frontal plane as well as reduced lumbar lordosis has been found to increase the risk of developing low back pain^17^, emphasizing the importance of preserving spinal flexibility and normal spinal curvature. However, obese adults have been found to have smaller lumbar flexion and lumbopelvic ratios in the frontal and horizontal plane than normal-weight people^9^. Nevertheless, it remains unclear whether spinal mobility is affected by obesity in children and adolescents, as studies to date that have examined this association between obesity in children, as well as adolescents and spinal mobility are lacking. Therefore, investigating this association in children and adolescents may help to better understand the spinal flexibility of children and adolescents with obesity and provide unique insights into the management of reduced spinal flexibility among this young population.

The aim of this study was to explore the association of obesity with spinal postures as well as spinal and hip mobility in children and adolescents and compare the parameters to those of the same age group with normal weight.

## Methods

The present study employed a cross-sectional design to explore the association of obesity with spinal posture as well as mobility and followed the Strengthening the Reporting of Observational Studies in Epidemiology (STROBE) guidelines.

### Participants

Obese and normal-weight children and adolescents as defined by the World Health Organization guidelines were recruited, with an age range between 6 and 18 years^18^. Participants suffering from past and present orthopaedic or neurological conditions, including limb length discrepancy, spina bifida, spinal deformities and surgeries determining physical disabilities and those taking anti-inflammatory drugs, were excluded from the study.

Normal-weight participants were recruited from elementary schools and sports clubs in the Canton of Zurich, Switzerland. Participants with obesity were recruited at the Division of Auxology, Istituto Auxologico Italiano, IRCCS, Piancavallo (VB), Italy, where they were hospitalized for a three-week multidisciplinary body weight reduction program. The study was approved by the Ethics Committee of the Istituto Auxologico Italiano (Milan, Italy; research project code: 01C124; acronym: PRORIPONATFIS) and the Ethics Committee of Zurich (BASEC-no. 2018-00979) and was in accordance with the Helsinki Declaration of 1975, as revised in 2008. The purpose and objective of the study were explained to each subject and his/her parents and written informed consent was obtained. The recruitment periods were between 26 October 2018 and 11 December 2019 for normal-weight children and adolescents and between 24 July 2020 and 21 April 2021 for children and adolescents with obesity.

### Measurements

#### Spinal and hip mobility

For children and adolescents with obesity, the measurements were performed on the 2nd–3rd day of hospitalization, between 8 and 10 a.m., at a comfortable ambient temperature (about 20 °C), by the same well-trained operator. The same condition of measurement (i.e. time, ambient) was adopted for the control group**.**

Spinal posture, as well as spinal and hip mobility, were measured using the Idiag M360 scan tool (Idiag, Fehraltorf, Switzerland)^19^. The Idiag M360, a non-invasive, radiation-free, reliable, computer-aided skin-surface device is designed to quantify the spinal posture and mobility through computer-assisted analysis and records angles of each vertebral joint and sacral inclination in the sagittal and frontal plane. Vertebral distances, positions of the vertebral bodies and the sacrum, the angles between them, as well as the mobility of each individual spinal segment are calculated during the recording. The device consists of two rolling wheels that measure vertebral distances and angles whilst they follow the vertebral spinous processes. The parameters measured by the rolling wheels are transferred radiographically by an analogue–digital converter to a personal computer. As the rolling wheels follow the spinous processes, data are sampled every 1.3 mm at a frequency of 150 Hz^20–22^. The validity of measuring segmental and global lumbar mobility using the Idiag M360 was determined in comparison with an X-ray examination, which, however, remains the gold standard for assessing spinal deformities^23–25^. Thoracic, lumbar and hip movement were measured in the sagittal and frontal plane when participants were asked to bend forward as far as they can into flexion followed by extension and lateral flexion from an upright standing position. Variables of interest for spinal mobility were thoracic and lumbar flexion, extension and lateral flexion, as well as hip flexion and extension. Spinal posture and mobility were measured once according to the protocol, also containing pictures illustrating the measurement procedures, provided by previous studies^20,21^. Furthermore, both operators who measured the spinal parameters in the normal-weight and obese participants had been adequately trained by the Idiag staff with the support of educational videos.

#### Reliability of the device

To ensure the reliability of the device in obese patients, a pilot test was conducted that included two repeated measurements of the same subject in ten adolescents with obesity. The standard error of measurement (SEM) of the spinal parameters measured in the present study was computed as SD·√(1 − ICC), where SD and ICC are the standard deviation and intraclass correlation coefficient of each spinal posture or mobility. We estimated the intrarater ICCs for the spinal postures, ranging from 0.86 to 0.94 and the SEM values ranged from 0.58^0^ to 0.70^0^. The intrarater ICCs for spinal mobility in the sagittal and frontal plane ranged from 0.87 to 0.98 and 0.57–0.80, respectively. The SEM values ranged from 0.29^0^ to 1.45^0^ for the sagittal plane measurements and 3.4^0^–3.5^0^ for the frontal plane measurements.

As far as the reliability of the device for spinal posture and mobility measurements in normal-weight children and adolescents is concerned, fair to high reliability was previously demonstrated^21^. The intrarater and interrater ICCs ranged from 0.61 to 0.96 and 0.70–0.93, respectively, while the SEM values ranged from 0.61^0^ to 13.18^0^^21^.

### Data processing

Each range of motion value for the variables of interest was determined by the difference between the range of motion values measured at the standing position and the end of motion ranges in the anatomical planes. The device measures the range of motion in each segment of the spine, i.e. five motion values for the lumbar spine and twelve for the thoracic spine. The total range of motion of the lumbar and thoracic spine was determined by the sum of the respective range of motion values. Hip range of motion was determined by the sacral inclination relative to the frontal plane.

### Statistical analysis

Descriptive statistics and inferential analyses were performed using the R version 3.6.0^26^. In descriptive statistics, mean values and standard deviations (SD) for participant characteristics, including age, sex, posture and mobility of the hip, thoracic and lumbar spine, as well as lumbar-hip ratio were determined. The Shapiro–Wilk test was used to check for data normality. The independent samples t-test for normally distributed data and the Wilcoxon’s test for non-normally distributed parameters were employed to compare demographic and anthropometric characteristics between the two groups (normal-weight and obese). The chi-square test was used for categorical variables. Age and sex-adjusted two-way analysis of variance (ANOVA) was performed for determining statistically significant postural and mobility differences between the normal-weight and obese groups. Pairwise post hoc tests were performed following the ANOVA tests using the software package “emmeans”^27^. A p-value of less than 0.05 was considered statistically significant.

## Results

**Figure 1:**
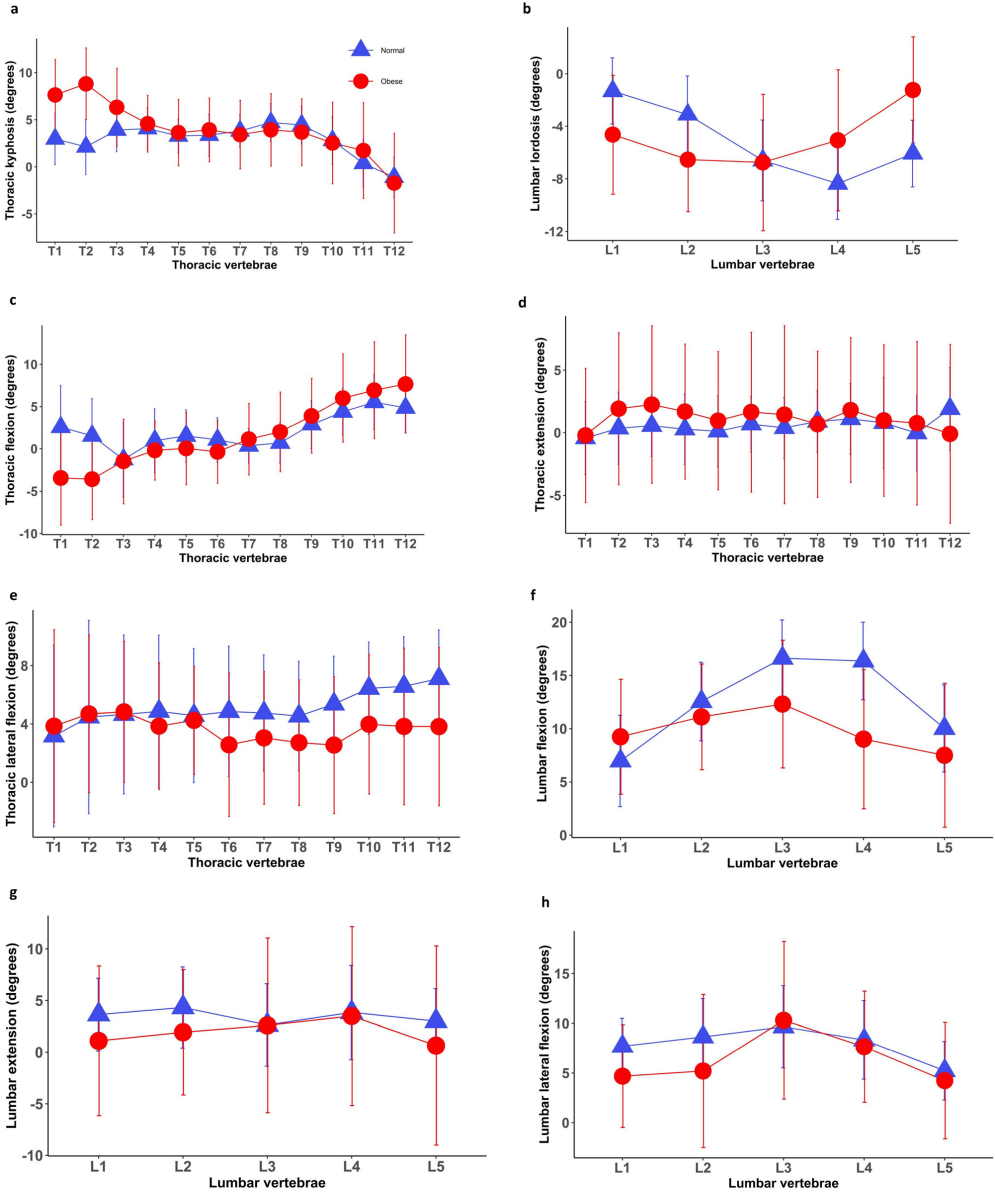
Posture and mobility of each individual spinal segment in the normal-weight and obese groups (mean ± standard deviations). *Normal* normal-weight group, *Obese* obese group.

A total of 109 children and adolescents with obesity and 90 normal-weight age-matched individuals were recruited for the study. The main demographic and anthropometric characteristics of the participants are presented in Table 1. The average age, height and sex ratio were not different between the two groups (p > 0.05). Segmental posture and mobility of the spine in the normal-weight and obese groups are presented in Fig. 1.

**Table 1:** Demographic and anthropometric characteristics in the normal-weight and obese groups (mean ± standard deviations).

Variables	Normal-weight children/adolescents (N = 90)	Obese children/adolescents (N = 109)	p-value
Age (years)	13.9 (3.4)	15.2 (2.0)	0.06^w^
Sex (female)	48%	58%	0.21^c^
Weight (kg)	51.7 (14.8)	95.8 (20.7)	< 0.0001^w^
Height (cm)	158.0 (16.3)	163.2 (10.0)	0.29^w^
BMI (kg/m^2^)	20.1 (2.4)	35.7 (5.7)	< 0.0001^w^

p-value—statistical significance computed by using Wilcoxon’s test ^w^ and the chi-square test ^c^ for a comparison between the two groups.

*BMI* body mass index.

### Association between obesity and spinal postures as well as the mobility of the spine and hip

The age- and sex-adjusted ANOVA test showed statistically significant differences in thoracic kyphosis and lumbar, thoracic and hip range of motion between the two groups (Table 2). Thoracic kyphosis was larger in the obese group than in the normal-weight group, whereas no statistically significant differences between the groups were found in the other spinal postures. The lumbar, thoracic and hip range of motion measured in the two anatomical planes was larger in the normal-weight group than in the obese group except for the thoracic extension. In the post hoc test, the largest differences observed in spinal and hip mobility between the two groups were in thoracic lateral flexion, followed by hip extension, which was 17.7^0^ and 14.8^0^, respectively, larger in the normal-weight group than in the group of obese children and adolescents (Table 2). The remaining lumbar and hip range of motion values were also larger in the normal-weight individuals compared to the obese individuals, except for the thoracic extension, which was 6.5^0^ larger in the obese participants. The lumbar-hip ratio was not different between the two groups, but a statistically significant difference was observed between the male and female individuals. The lumbar-hip ratio was smaller in the female participants than in the male subjects (p < 0.0001).

**Table 2:** Participants’ characteristics, spinal postures, lumbar, thoracic and hip mobility differences between the normal-weight and obese groups (mean ± standard deviations).

Variables	Normal-weight children/dolescents (N = 90)	Obese children/adolescents (N = 109)	Differences in spinal posture and mobility (CI)	p-value
**Spinal postures**				
Thoracic kyphosis (Th1-12)	35.2 (9.1)	48.2 (10.6)	− 13.0 (− 15.8 to − 10.1)	< 0.0001
Proximal thoracic kyphosis (Th1-6)	20.0 (6.5)	34.6 (7.5)	− 14.6 (16.7 to 12.6)	< 0.0001
Distal thoracic kyphosis (Th7-12)	15.2 (6.7)	13.6 (11.2)	1.6 (− 1.1 to 4.4)	0.23
Lumbar lordosis	25.8 (7.7)	23.8 (11.4)	2.0 (− 0.9 to 4.8)	0.18
Sacral kyphosis	14.5 (7.4)	13.1 (9.8)	1.4 (− 1.1 to 4.0)	0.26
**Spinal mobility**				
Thoracic (^0^)				
Flexion	24.3 (11.0)	19.3 (14.9)	5.0 (1.2–8.8)	0.01
Extension	7.0 (14.2)	13.5 (16.5)	− 6.5 (− 11.6 to − 2.9)	0.005
Lateral flexion	61.9 (24.4)	44.2 (18.3)	17.7 (11.6–23.8)	< 0.0001
Lumbar (^0^)				
Flexion	62.0 (8.4)	49.9 (14.6)	12.1 (8.7–15.5)	< 0.0001
Extension	17.0 (8.7)	9.9 (20.4)	7.1 (3.1–12.2)	0.003
Lateral flexion	40.5 (12.6)	31.4 (13.2)	9.1 (5.5–12.8)	< 0.0001
**Hip mobility**				
Hip (^0^)				
Flexion	46.5 (16.5)	36.0 (14.8)	10.5 (6.3–14.6)	< 0.0001
Extension	17.6 (13.9)	2.8 (14.8)	14.8 (10.6–19.0)	< 0.0001
Lumbar-hip ratio	0.57 (0.1)	0.58 (0.2)	− 0.01 (− 0.04 to 0.03)	0.81

p-value—statistical significance for comparison between the two groups.

*CI* confidence interval.

Lumbar–hip ratio (during trunk flexion in the sagittal plane) = lumbar range of motion/(lumbar range of motion + hip range of motion).

## Discussion

The purpose of the current study was to examine the association of obesity with spinal posture and mobility in children and adolescents, with the key finding being that obesity is associated with alterations in spinal and hip mobility, as well as some spinal postures. Obesity in children and adolescents was associated with increased thoracic kyphosis and thoracic extension but decreased mobility in thoracic flexion and lateral flexion, lumbar flexion, extension and lateral flexion and hip flexion and extension. Obesity appears to have an influence on increased thoracic kyphosis, which in turn may require increased thoracic extension but no effect on lumbar and sacral postures.

Obesity was associated with increased thoracic kyphosis, but no significant association was observed with lumbar and sacral postures. Although studies determining thoracic kyphosis in children and adolescents with obesity using a skin-surface device are lacking, the average kyphosis in the normal-weight participants determined at 35^0^ in the current study was similar to estimates for thoracic kyphosis defined by a previous study involving subjects with a mean age of 10.6 years^21^. The results from the current study are also consistent with previous study findings in relation to the association between obesity and thoracic kyphosis. A retrospective comparative cohort study of 70 nonscoliotic adolescents and 1551 adolescent idiopathic scoliosis patients that explored the relationship between BMI (body mass index) and thoracic kyphosis, found that an increased BMI was associated with increased thoracic kyphosis in both individuals with and without spinal deformities^28^. The authors suggested that excess weight may hinder anterior vertebral growth by increasing compressive load on the growth plates of the vertebrae, potentially resulting in increased kyphosis. Additionally, they highlighted that this association needs to be taken into consideration when correcting sagittal plane deformities. In the current study, the thoracic spine was further examined segmentally (proximal T1_6 and distal T7_12) in relation to obesity. An increase in proximal thoracic kyphosis was associated with obesity, whereas no significant association was observed with distal thoracic kyphosis, thus suggesting that obesity may contribute more to alterations in the sagittal posture of the upper thoracic spine (T1_6) than the lower thoracic spine. Increased thoracic kyphosis appears to be correlated with some musculoskeletal conditions. For example, a previous case–control study found that increased thoracic kyphosis was associated with the shoulder impingement syndrome^29^, implying the importance of taking into account thoracic kyphosis in the management of the shoulder impingement syndrome. Additionally, extensive knowledge of thoracic posture and mobility is crucial for an effective strategy in the management and prevention of thoracic disorders, as well as the evaluation of rehabilitation outcomes^11^. A previous systematic review exploring the shape and mobility of the thoracic spine in asymptomatic individuals found that thoracic kyphosis increased with ageing, an increase of approximately 3 degrees per decade^11^, implying that alterations in thoracic kyphosis tend to increase over time. Thoracic kyphosis also appears to change between sitting and standing positions^30,31^. For example, a comparative study of 58 individuals with a mean age of 23 years found that thoracic kyphosis decreased from standing to sitting due to the forward displacement of the sagittal vertical axis, likely as a result of the change in the centre of gravity^30^. The results from the current study provide important information about the characteristics of the thoracic spine in obese children and adolescents and unique insights into obesity-related mechanical challenges such as excess weight in the anterior part of the trunk that the thoracic spine must withstand. These findings also imply the importance of taking obesity into account when assessing thoracic kyphosis and when treating or correcting thoracic kyphosis in this paediatric population.

Lumbar and sacral postures were not different between obese and normal-weight children and adolescents in the current study. The result was in line with previous cross-sectional studies that analysed the influence of the BMI on lumbar and pelvic postures determined by using X-rays^32,33^. A cross-sectional study of 200 adult participants categorized into normal-weight, overweight and obese groups that investigated the relationship between obesity and spinopelvic parameters, including lumbar lordosis, pelvic tilt and sacral slope, showed no statistically significant differences in the parameters across the three different BMI groups^33^. Another cross-sectional study of 89 elderly females analysing the influence of the BMI on lumbar and pelvic postures found that lumbopelvic postures appear to be unaffected by the BMI^32^, suggesting that the BMI may not influence lumbopelvic postures, regardless of age. The findings of the current study also suggest that obesity appears to contribute less to alterations in lumbopelvic postures than thoracic kyphosis. However, studies that explore the association between obesity and lumbopelvic postures in children and adolescents are lacking, suggesting that more research is needed to confirm the findings from the current study.

All the spinal movement measured in the sagittal and frontal plane were significantly different between obese and normal-weight children and adolescents. Alterations in spinal mobility are often associated with musculoskeletal conditions, particularly low back pain^12,13^. Regarding the thoracic spine, it was found that thoracic flexion and lateral flexion were lower in obese patients than in normal-weight individuals, suggesting that obesity may contribute to reduced thoracic flexibility in this paediatric population. Although studies analysing the association between obesity and spinal mobility in children and adolescents are lacking, the previous systematic review that included 45 studies of adult populations showed that obesity was associated with reduced thoracic movement^11^, which was consistent with the findings from the current study. However, the thoracic extension was larger in obese children and adolescents than in normal-weight individuals, which could potentially be specific to this age group. This could also be explained by increased thoracic kyphosis found in obese individuals, which in turn may require an increased thoracic extension. Thus, the different thoracic positions in standing (mid position) between the normal-weight and obese groups may explain the range of motion differences in the sagittal plane. These range of motion differences may not be related to their flexibility but rather to the different thoracic positions in standing. These results may have important implications regarding the assessment of thoracic movement and strategies aiming to improve thoracic mobility in children and adolescents.

Lumbar flexion, extension and lateral flexion were found to be smaller in obese patients than in normal-weight individuals. Studies examining the relationship between obesity and lumbar mobility in adolescent children are also lacking to date. Nevertheless, several studies have examined this association in adult populations. The reduced lumbar flexion observed in the obese population in the current study was in line with a previous cross-sectional study of 18 obese and normal-weight adults comparing lumbar movement measured by using a Vicon motion capture system^9^. Another cross-sectional study that compared lumbar mobility determined by using a back range of motion instrument, the BROM II, between 20 obese and 20 non-obese males, found no effect of obesity on lumbar flexion, showing some inconsistencies with the association between obesity and reduced lumbar flexion in adult populations^34^. Nevertheless, this study found that obesity was associated with reduced lumbar extension and lateral flexion, which was consistent with our findings. Reduced lumbar movement associated with obesity appear to be explained by excess adipose tissues obstructing vertebral intersegmental mobility^34^. Additionally, reduced lumbar mobility is often associated with low back pain^12,13^. For example, a meta-analysis of prospective studies showed that a restriction in the lumbar range of motion in the frontal plane increases the risk of developing low back pain^17^. Overall, the findings suggested that obesity appears to contribute to reduced lumbar mobility in the sagittal and frontal plane, which may be taken into account when developing strategies designed to prevent a reduction in lumbar mobility-associated obesity and promote lumbar flexibility in this paediatric population.

Obesity was found to be associated with reduced hip flexion and extension, whereas no associations were observed with the lumbar–hip ratio. Hip flexion and extension were approximately 10.5 (6.3–14.6) and 14.8 (10.6–19.0) degrees smaller in the obese subjects, respectively, suggesting that obesity may contribute more to a reduction in hip extension than to a hip flexion range of motion. Although hip flexion was found to be smaller in obese children and adolescents, no statistically significant differences were observed in the lumbar-hip ratio between the obese and normal-weight populations. This may be explained by a decrease in both the lumbar and hip flexion range of motion, as the lumbar-hip ratio was determined by the proportion of these two parameters. However, a statistically significant difference in the lumbar-hip ratio was observed between the male and female individuals, implying that gender potentially influences the lumbar-hip ratio more than obesity. These findings highlight that obesity may contribute more to alterations in the hip range of motion, particularly hip extension, than the lumbar-hip ratio in children and adolescents.

The main limitation of the current study is the cross-sectional design, which cannot provide evidence on whether the nature of the association between obesity and alterations in spinal and hip mobility is causal. The current study examines spinal posture and the mobility of the spine and hip only in children and adolescents, thus hampering a generalization of the results to people outside this age range. Spinal mobility, particularly lumbar extension, in individuals with obesity, could be underestimated due to a greater percentage of soft tissue, as we used a skin surface device in the current study. Spinal mobility (range of motion) was determined by the change in angles between the static positions defining the starting point and the end of the spinal range of motion, suggesting that the range of motion values estimated in the current study could not be generalized to a dynamic range or motion or dynamic flexibility. Spinal parameter differences found in the current study between normal-weight participants recruited in elementary schools and sports clubs and the hospitalized children and adolescents with obesity may not be generalized to comparisons of these parameters between normal-weight children and adolescents who do not engage in sports activities and non-hospitalized children and adolescents with obesity.

In conclusion, alterations in spinal postures, as well as spinal and hip mobility, were associated with obesity in children and adolescents. Obesity was associated with increased thoracic kyphosis and thoracic extension but reduced spinal and hip mobility, including thoracic flexion and lateral flexion, lumbar flexion, extension and lateral flexion, as well as hip flexion and extension except for the thoracic extension. Obesity appears to contribute to increased thoracic kyphosis, which in turn may require increased thoracic extension but has no effect on lumbar and sacral postures or the hip-to-lumbar ratio. These findings provide important information about the characteristics of the spine in obese children and adolescents and unique insights into obesity-related mechanical challenges that the spine has to withstand. These results also imply the importance of taking into account obesity in the assessment of spinal postures, as well as strategies designed to improve spinal and hip mobility in children and adolescents.

## Data Availability

The data presented in this study are available from the corresponding author upon reasonable request.
